# A comparison of zircon U-Pb age results of the Red Clay sequence on the central Chinese Loess Plateau

**DOI:** 10.1038/srep29642

**Published:** 2016-08-19

**Authors:** Hujun Gong, Junsheng Nie, Zhao Wang, Wenbin Peng, Rui Zhang, Yunxiang Zhang

**Affiliations:** 1 Institute of Cenozoic Geology and Environment, State Key Laboratory of Continental Dynamics, Department of Geology, Northwest University, Xi’ an 710069, China; 2 Key Laboratory of Western China’s Environmental Systems (Ministry of Education), College of Earth and Environmental Sciences, Lanzhou University, Lanzhou 73000, China

## Abstract

Single grain zircon U-Pb geochronology has demonstrated great potentials in extracting tectonic and atmospheric circulation signal carried by aeolian, fluvial, and fluviolacustrine sediments. A routine in this sort of studies is analyzing 100–150 grains and then compares zircon U-Pb age spectra between the measured sample and the potential sources. Here we compared the zircon U-Pb age results of the late Miocene-Pliocene Red Clay sequence of two neighboring sites from the Chinese Loess Plateau where similar provenance signal is expected. Although the results from the 5.5 Ma sediment support this prediction, the results from the 3 Ma sediment at these two sites differ from each other significantly. These results emphasize the importance of increasing analysis number per sample and combining the zircon U-Pb geochronology with other provenance tools in order to get reliable provenance information.

Provenance analysis has been widely used to solve diverse geological problems, such as river dispersal pattern, formation age of seaway, dust transport routes and atmospheric circulation pattern, and mountain exhumation history^1^,^2^,^3^,^4^,^5^,^6^,^7^,^8^,^9^,^10^,^11^,^12^,^13^,^14^,^15^. Traditional provenance analysis relies on bulk method such as Pb, Nd and Sr isotope^1^,^15^,^16^,^17^,^18^,^19^,^20^,^21^, but recent technique progresses made single-grain methods popular because single-grain techniques allow recognition of multi- source signal which will be lumped together by the bulk methods^14^,^22^. Particularly, zircon U-Pb geochronology played key roles in provenance analysis recently because by recognizing multi- age populations, this method often allows clearly pinpointing multi- source regions^3^,^23^,^24^. One routine^5^,^7^,^11^,^25^,^26^,^27^,^28^ in using zircon U-Pb geochronology as a provenance tracing tool is to measure 100–150 grains per sample. Despite one research^29^ demonstrates that more than 500–1000 grains are needed to make a quantitative comparison, it is still under the impression of many workers that qualitative comparison of U-Pb age spectra similarity is useful to infer provenance with 100–150 measurements per sample.

Here we present an example of comparing zircon U-Pb age results for two Red Clay sites (Lingtai and Chaona) within ~50 km on the central Chinese Loess Plateau (CLP). Recent backtrace trajectory experiments^30^ demonstrate that these central CLP sites should have similar provenance signal for stratigraphically equivalent or approximately equivalent samples. However, we show that the zircon U-Pb age results based on 100–150 analysis per sample are not completely consistent with this prediction.

## Materials and Method

**Figure 1:**
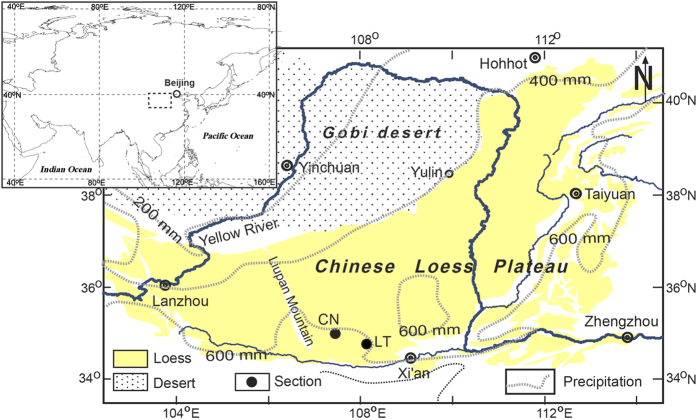
Map showing the Chinese Loess Plateau and location of the study sites. The main map corresponds to the area within the rectangle in the index map. Revised from Nie *et al.* (2013)^41^.

Lingtai and Chaona are located in the central CLP with similar annual mean temperature and precipitation (Fig. 1). The Red Clay sequence in the Lingtai and the Chaona site is ~130 m and ~125 m, with a basal age of ~7 Ma and ~8 Ma, respectively^31^,^32^.

Four Red Clay samples and one underlying sandstone sample were obtained from the Lingtai section. The samples were taken at depths of 190, 250, 280, and 305 m, with magnetostratigraphic ages of about 3, 5.5, 6 and 7 Ma (hereafter LT-3 Ma, LT-5.5 Ma, LT-6 Ma and LT-7 Ma), respectively^31^. In the laboratory, the samples were ground and then moved across a water-shaking table to remove clay. The remaining material was subjected to heavy liquid separation to concentrate the heavy minerals, and subsequently, this fraction was run through a Frantz magnetic separator to separate zircons. The zircons were mounted and analyzed at Northwestern University, China, following standard procedures^33^. ^206^Pb/^238^U ages and ^207^Pb/^206^Pb ages were used for ages younger and older than 1000 Ma, respectively. We report the ages within 30% discordance and 10% negative discordance. The discordance was calculated based on (^207^Pb/^206^Pb-^206^Pb/^238^U)/^207^Pb/^206^Pb*100 and (^207^Pb/^235^U-^206^Pb/^238^U)/ ^207^Pb/^235^U*100 for ages younger and older than 1000 Ma, respectively.

Four Chaona Red Clay samples’ zircon U-Pb ages were already published^34^. We included these samples for comparison with the Lingtai data. There are two Chaona samples (one at 3 Ma and the other at 5.5 Ma; here after CN-3 Ma and CN-5.5 Ma) having equivalent depositional age as the Lingtai samples. The other two samples with depositional ages of 4 Ma and 8 Ma (hereafter CN-4Ma and CN-8 Ma) are within 1 Myr range of LT-3 Ma and LT-7 Ma. This is the first time that zircon U-Pb results are compared at this close distance for the Red Clay sequence.

We compared the zircon U-Pb age results of the Lingtai and Chaona samples based on visual comparison and the non-matrix multi-dimensional scaling (MDS) technique. This technique is originally based on the Kolmogorov–Smirnov (K–S) statistical method and works in a similar way to principal component analysis in which the distance resembles the similarity^35^. Because K-S statistical test emphasizes the maximum difference of the cumulative distribution function between two datasets but the Chinese loess have multiple populations of ages, Che and Li (2013) replaced the K-S test with difference between Kernel density estimation of two datasets, resulting in an integrated consideration of all age populations^16^. We reported the MDS results based on both the K-S test and the Kernel density estimation difference.

## Results

**Figure 2:**
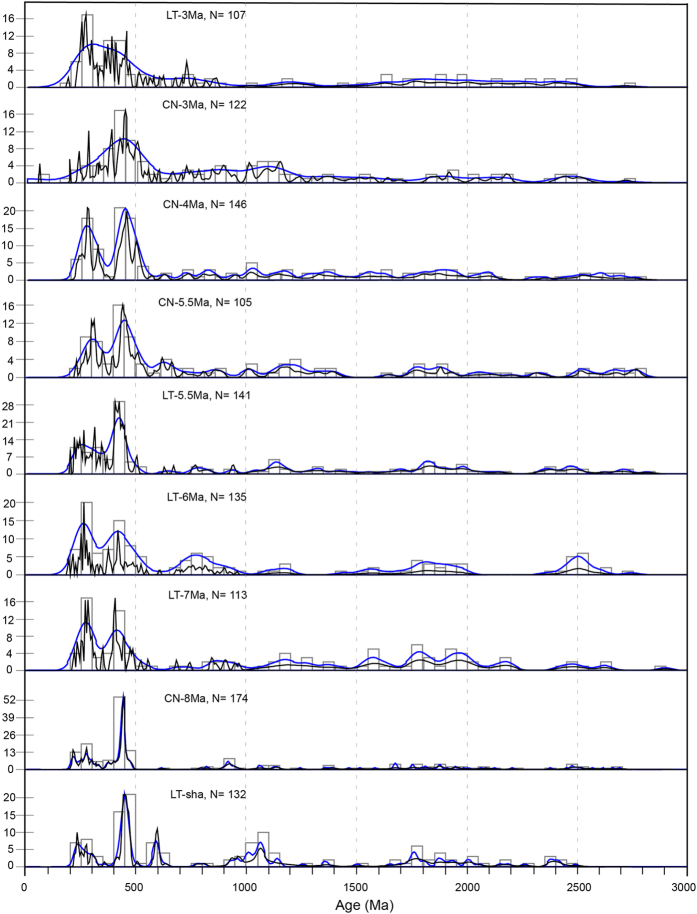
Distribution of detrital zircon U-Pb ages for Lingtai and Chaona Red Clay samples and one underlying sandstone sample. Black and blue lines are normalized probability density (PDP) and Kernel Density Estimation (KDE) plots^42^, respectively; and the open rectangles are age histograms. Samples are arranged in stratigraphical order.

Figure 2 illustrates the visual comparison results (Supplementary Dataset 1). The 500–400 Ma age population is the most abundant age group for the Lingtai sandstone sample (LT-Sha), which also has ages within the ranges of 300–200, 650–550, 1100–900 and 2500–1600 Ma. The Chaona lowermost Red Clay sample (CN-8 Ma) is dominated by the 500–400 Ma age group, and the next most abundant age group is 300–200 Ma. However, in contrast to the LT-Sha sample, the CN-8 Ma sample has few Precambrian ages. The dominant 500–400 Ma age population, and minor population of Precambrian ages of sample CN-8 Ma, are similar to the zircon U-Pb age distribution pattern of fluviolacustrine samples from the Qaidam Basin^5^,^34^.

LT-7Ma and LT-6Ma have a visually similar age distribution pattern. They are dominated by 500–400 Ma and 300–200 Ma components, with the latter comprising a slightly lower proportion than the former. In addition, both samples have ages of 900–600 Ma, 1500–1000 Ma, 2200–1500 Ma and 2700–2300 Ma.

The two 5.5 Ma samples from Lingtai and Chaona (LT-5.5 Ma and CN-5.5 Ma) are visually similar. In contrast to LT-7 Ma and LT-6 Ma, they have a larger 500–400 Ma population component compared to the 300–200 Ma component. The 4 Ma sample from Chaona (CN-4 Ma) has roughly equal contributions from the 500–400 and the 300–200 Ma groups. The two 3 Ma samples from Lingtai and Chaona (LT-3 Ma and CN-3 Ma) contrast sharply with each other, with LT-3 Ma dominated by the 500–400 Ma group and CN-3Ma by the 300–200 Ma group.

**Figure 3:**
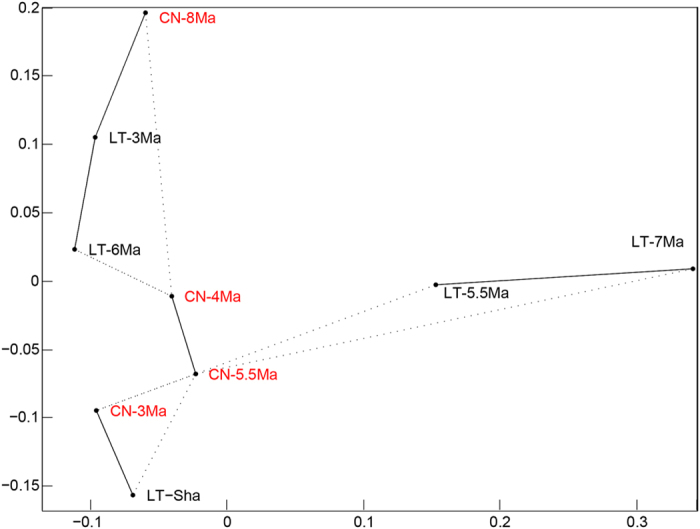


**Figure 4:**
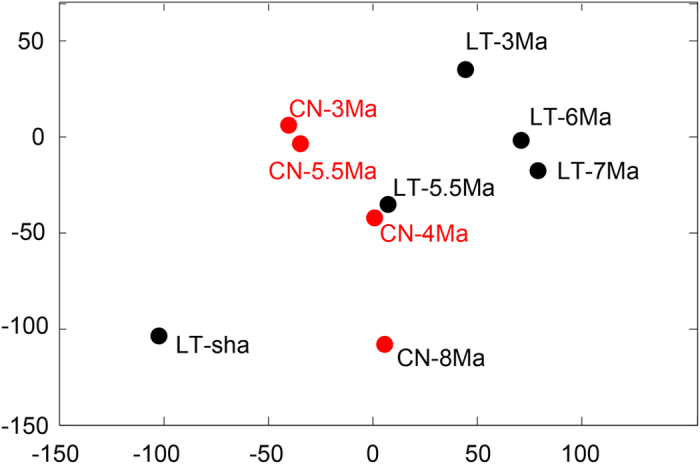
Kernel density estimation-based MDS plot for samples in fig. 2.

In contrast with the visual comparison, the K-S-based MDS comparison reveals different patterns (Fig. 3). First, the LT-Sha sample is far apart from CN-8Ma but instead shows similarity with CN3Ma. Second, LT-7 Ma and LT-6 Ma are also far apart. Third, CN-8 Ma is close to LT-3 Ma instead to LT-Sha. The Kernel density estimation-based MDS plot shows different patterns from the K-S-based MDS plot (Fig. 4). First, the LT-Sha and CN-8 Ma are close to each other. Second, LT-7 Ma and LT-6 Ma are close. Third, CN-8 Ma is not close to LT-3 Ma. Obviously, the Kernel density estimation-based MDS plot is more consistent with the visual comparison result. In any case, the MDS results reveal that the oldest and the youngest samples are located at the periphery of the plots and the intermediate-aged samples are located in the center, and the LT-3 Ma and CN-3 Ma are not similar with each other (Figs 3 and 4).

## Discussion

Vermeesch (2004) demonstrated that in order for age population with >5% fraction is detected in the zircon U-Pb result at the 95% confidence level, 117 ages should be reported. Since then, many workers have followed this recommendation and measured 100–150 grains when using zircon U-Pb geochronology to constrain provenance. However, in such practice, many workers tend to compare similarity between the age spectra between different samples to infer source-sink relationships, with the assumption that at least at a qualitative sense, this comparison is valid.

The age models for both the Lingtai^31^ and Chaona^32^ sections are based on paleomagnetic dating–via correlation of the observed polarity sequence with the Geomagnetic Polarity Time Scale^36^ and thus permitting their direct comparison within a common geochronological framework. Although the similar zircon U-Pb age spectra for the 5.5 Ma samples between Lingtai and Chaona support the practice of inferring provenance information from comparing zircon U-Pb age spectra similarity when the reported ages are between 100 and 150, the strikingly different age spectra for LT-3 Ma and CN-3 Ma highlight the need to increase analysis number per sample to warrant a robust comparison of similarity. It is worth noting that the visual comparison reveals different similarity pattern compared to the quantitative comparison based on the K-S-based MDS technique (Fig. 3) but more similar to the Kernel density estimation-based MDS result (Fig. 4), so presenting MDS results based on both techniques would be complementary. We also note that Licht *et al.* (2016) demonstrate that the MDS statistical technique is also affected by the number of zircon grains.

The reason for not reporting more than 150 zircon ages per sample in zircon U-Pb dating are multiple, such as labor and expenses^25^. However, QEMSCAN-based (Quantitative Evaluation of Minerals by Scanning Electron Microscopy) heavy mineral analysis can provide supporting evidence for the zircon U-Pb data^37^. This technique allows rapid recognition thousands of heavy minerals within seconds. Therefore, a practical way of performing provenance analysis in future efforts is a combination of the QEMSCAN heavy mineral analysis with the zircon U-Pb geochronology technique. At noted in several workers, reliably extracting provenance information from sediments is often not straightforward: issues like recycling, fertility of certain mineral types to source terrains can significantly bias the story based on a single technique^7^,^38^,^39^,^40^. Therefore, an integrated strategy is safest to reliably reconstruct provenance history.

## Additional Information

**How to cite this article**: Gong, H. *et al.* A comparison of zircon U-Pb age results of the Red Clay sequence on the central Chinese Loess Plateau. *Sci. Rep.* **6**, 29642; doi: 10.1038/srep29642 (2016).

## Supplementary Material

Supplementary Dataset 1
